# Childhood leukaemia and lymphoma: African experience supports a role for environmental factors in leukaemogenesis

**DOI:** 10.3332/ecancer.2014.478

**Published:** 2014-11-06

**Authors:** Christopher KO Williams, Letizia Foroni, Lucio Luzzatto, Idris Saliu, Arthur Levine, Mel F Greaves

**Affiliations:** 1 Department of Haematology, College of Medicine, University of Ibadan, Ibadan, Nigeria; 2 Hematology Oncology Consultancy, Port Angeles, WA 98362, USA; 3 FHCRC/UW Centre for AIDS Research (CFAR), Seattle, WA 98104, USA; 4 Department of Haematology, Royal Postgraduate Medicine School, London W12 ONN, England; 5 Viral Epidemiology Branch, Landow Building 3C19, National Cancer Institute, Bethesda, MD 20205, USA; 6 Chester Beatty Laboratories, London SW3 6JB, England

**Keywords:** childhood leukaemia, lymphoblastic, environmental factors, chloroma, leukaemogenesis, socio-economic, lifestyle

## Abstract

Major differences exist in the nature of leukaemia and lymphoma in low-income African children compared to those in the high-income countries. These include the absence of the peak incidence of acute lymphoblastic leukaemia (ALL) in under-five-year olds that characterizes the disease in high-income countries. Conversely, chloroma association with acute myelogenous leukaemia (CA-AML/AMML) and Burkitt’s lymphoma (BL) are rare in the high-income countries. This report describes clinical and laboratory as well as epidemiological features of childhood leukaemia and lymphoma reported betwen 1982 and 1984 in the city of Ibadan, Nigeria. The observed pattern of distribution of childhood haematological malignancies in the city is more consistent with the observations of Ludwik Gross’s experiments on environmental influences, such as malnutrition and infections, animal leukaemogenesis, and mirroring the consequences of the primordial pressures that have shaped human genetics and pathophysiology.

## Introduction

Paediatric neoplasia, though relatively rare, is important for the understanding of oncogenesis [1]. Studies of the African childhood lymphoma, otherwise known as Burkitt’s lymphoma, in the 1960s and 1970s, led to the appreciation of the role of environmental factors in oncogenesis [2, 3]. Furthermore, the unique chromosomal aberrations that are associated with this disease led to the understanding of the role of molecular events in cancer aetiology [4]. Acute lymphoblastic leukaemia has served as a model for the development of curative cancer management [5, 6].

Though the incidence of childhood cancers is similar throughout the world, major differences exist in many parts of the world with respect to childhood haematological malignancies [7, 8]. For example, the features of childhood leukaemia and lymphoma in low-income African countries are different from those of children in the high-income countries. These differences include: the rarity of leukaemia below the age of 5 in the former, unlike the peaking of the incidence of the disease in the age group in the latter [9–12]; the high incidence of Burkitt’s lymphoma (BL) in the former, and its rarity in the latter population. Although the incidence of acute myeloid leukaemia in children varies little, there is considerable variability in its clinical manifestations in various parts of the world. The disease, for instance, is frequently associated with solid-tumour formation (chloromas) in the developing countries, leading to its being designated as ‘chloroma-associated acute myeloid leukaemia (CA-AML/AMML)’ [13, 14], with clinical features that make it almost indistinguishable from the more common BL.

Several factors have been implicated in the past in the aetiology of childhood ALL [15]. In the study of the geographical distribution of leukaemia subtypes, Greaves and his colleagues [16] observed a striking difference in the proportion of the common acute lymphoblastic leukaemia (c-ALL) subtype between the affluent population of the United States, Europe, and South Africa and the less affluent populations of Nigeria and South Africa. Some of these observations led Greaves to postulate the hypothesis of ‘aberrant immune response’ to common infection(s) as a plausible aetiological mechanism for ALL [15].

The objective of this report is to seek explanation about the variations in the frequency of paediatric haematological cancers in the light of the varying lifestyles in communities.

## Patients and methods

The patients included in this study were seen and assessed clinically at the University College Hospital (UCH), Ibadan, Nigeria. Initial laboratory diagnostic tests included a complete blood count (CBC) including differential leucocyte count on a Romanovsky-stained blood film. Bone marrow aspirates were routinely obtained in all cases of acute leukaemia and lymphoma and films prepared therewith were routinely processed with May Gruenwald Giemsa stain, and, in cases of acute leukaemia, with periodic acid Schiff (PAS) and Sudan Black stains. Haematoxylin Eosin-stained tissue sections were routinely obtained for the diagnosis of malignant lymphoma. Cells for immunophenotypic characterization were obtained from the tissues involved that could be conveniently sampled. Thus, heparinized peripheral blood was obtained in the cases of acute or chronic leukaemia with a total WBC in excess of 20.0×109/L, while in other cases heparinized bone marrow blood was utilized. In the cases of malignant lymphoma, the samples were obtained in the forms of cerebro spinal, ascitic or pleural fluids, biopsy of enlarged lymph nodes, or the involved viscera. When necessary, such samples were teased to release an adequate quantity of cells for the procedures.

The laboratory procedures of immunophenotypic characterization were performed according to the protocol of the International Study of Cell Markers in Leukaemias and Lymphomas as outlined by Greaves *et al* [16], using a panel of first generation reagents including: J 5 [17] and AL2 [18], both anti CALLA; DA 2, an anti HLA DR [19], WT 1, an anti T [20] and OKT11a, an anti E rosette receptor [21]; and My906, an anti myeloid [22, 23] monoclonal antibody. A large number of heterologous anti sera, such as anti Ig, anti kappa, anti lambda, and anti Tdt (terminal deoxynucleotidyl transferase) were also included in the panel [24]; anti T subset murine monoclonal antibodies, including OKT3, OKT4, OKT6, and OKT8 [25, 26]. The binding of the monoclonal antibodies to target cells was determined by indirect immunofluorescence with fluoresceinated goat anti mouse IgG (in case of anti Tdt: rabbit anti mouse IgG) or by direct immunofluorescence in case of detection of cell-surface immunoglobulin, using a Leitz Ortholux II fluorescence microscope with incident illumination.

The capability of some T lymphocytes and B lymphocytes to form rosettes with sheep [27] and mouse red blood cells [28], respectively, was also used in the process of characterization.

## Criteria for subtype characterization

### Acute lymphoblastic leukaemia

Only cases of acute leukaemia that did not react with the myeloid monoclonal My906 (CD33) were diagnosed as ALL. The subtypes of ALL were defined according to the previously published algorithm for the interpretation of immunophenotypic patterns observed in the International Study of Cell Markers in Leukaemias and Lymphomas [29], however, with some modifications as outlined by Borowitz [30]. The subsets were defined as follows: common-ALL: CALLA+, DR+, T (WT1/E/T11)-, smIg-, Tdt+; null-ALL: CALLA-, DR+, T (WT1/E/T11)-, smIg-, Tdt-; T-ALL: cALLA+/- DR+/- T (WT1/E/T11)+, smIg-, Tdt+; B-ALL: CALLA-, DR+, Tdt-.

### Gene rearrangement studies

Samples of mononuclear cells from a few of the patients stored at −80 °C for several weeks were shipped to London, England, where they were studied for the evidence of gene rearrangement using a methodology that has previously been described [31].

### Clustering studies

For the purpose of studying the association of leukaemia subtypes with lifestyles, the city of Ibadan was sub-categorized into three zones depending on the lifestyle and social structure of the areas—Zone 1: the indigenous, old, and largely unplanned area, inhabited mainly by indigenes of the city, most of them are farmers, petty traders, and semi-skilled labourers. Environmental sanitation in this zone is very poor, and literacy is low. The average annual income is also very low (less than US$1,000 per year). Zone 2: non-indigenous high-density area inhabited by mixed population of business people, petty traders, professionals, skilled and unskilled labourers from various parts of the country, mainly from the neighbouring Yoruba-speaking States of the Federation of Nigeria. The level of education is generally higher than in the indigenous areas. The average annual income is intermediate between those of Zones 1 and 3. Zone 3: low/medium population density areas, consisting largely of parkland estates and predominantly inhabited by business people, academics, and professionals. The literacy rate is high; average annual income is the highest of all the three zones, and the lifestyle is generally comparable to that of suburban Western Europe or United States. The average annual income of the inhabitants of Zone 3 areas is above $10,000.

Each study subject was assigned to one of five socio-economic status (SES) groups depending on the level of education and occupation:

SES Group 1: Highly educated, senior public officers, business executives (estimated annual income: $10,000 or more)

SES Group 2: Post-secondary school educated; middle-level public officers (estimated annual income: $5,000–$9,000)

SES Group 3: Post-primary school educated, lower-level public officers or institutional staff and skilled handworkers (estimated annual income: $2,000–$3,500)

SES Group 4: Primary school educated and unskilled hand-workers (estimated annual income: $1,000–$1,500)

SES Group 5: Illiterate peasant farmers and petty traders (estimated annual income: less than $1,000)

The population sizes of the various SES groups of the residents of Ibadan city were projected from the most recently available census figures of 1963, assuming a uniform growth rate of between 2.5% and 5.0% for all five SES groups among children and adults. The distribution of the total estimated population into the various socio-economic groups was based on the studies of Odebiyi [32] and Onibokun *et al* [33]. The reports of these studies suggested that individuals of low-, medium-, and high- socio-economic groups in the city constituted 75%, 12.5%, and 12.5%, respectively. There was no useful information in deriving the sizes of the population of the three categories of residential zones.

Furthermore, the calculation of children and adult population within the various socio-economic groups rested on the assumption that the World Bank [34] estimation of 47% and 53% for children (aged less than 15 years) and adults (aged 15 and above) applied uniformly for all socio-economic groups.

Spatial and temporal clustering of a particular disorder was determined to have taken place, respectively, if at least two cases of the disorder occurred in individuals resident within a distance of 2 km of one another and within a time span of 6 months.

## Results

Eighty-four cases of malignant lymphoproliferative disorders and leukaemia were immunophenotyped at the Department of Haematology, University College Hospital (UCH), Ibadan, Nigeria between September 1982 and December 1984. They included 17 cases of childhood (age <15 years) ALL, 20 of adult (age ≥15 years) ALL, 6 cases of AML, 23 of CLL and 18 of non Hodgkin’s lymphoma. Specimens were available from 32 of the immunophenotyped individuals for gene rearrangement studies, which were subsequently carried out in June 1986. Of these, only 19, which were suitable for DNA extraction could be studied for gene rearrangements. The categorization of the disease subtype was based on immunophenotypic characteristics (see above) as well as gene rearrangement features (Table 1).

### International comparative studies

A comparison of the pattern of ALL subtypes among Nigerians as observed in this study with the reported observations in USA (including Caucasian and African–American patients), UK, and Malaysia is provided in Table 2. C-ALL was least frequent (22.2%) among Nigerian children, next to the frequency of this condition among Malaysian and African American children. T-ALL was with 38.9% the most frequently encountered ALL subtype among Nigerian children. The frequency of c-ALL among adult Nigerian ALL patients (40%) was also lower than the observation among white adult Americans (50.7%) and adult UK patients (54.8%), but the differences were much less striking than the observations among children. Again, T-ALL was with 55% the most common subtype of ALL among adult Nigerian patients. This was much higher than the observation among adult white Americans (28.8%) and adult UK patients (11.9%).

An estimate of the incidence rates of ALL and its subtypes in Nigerian, British and American children are outlined in Table 3. This is based on a previous comparative study of the pattern of ALL in the three countries, using Ibadan, Nigeria data [14], Birch *et al* 1980, [35] and Young *et al* 1975 [36] as well as those of Greaves *et al* 1981 [37], Royston *et al* 1983 [38] and Bowman *et al* [39]. Although the incidence of ALL among Nigerian children was less than a third of those of the Caucasian children of UK and USA, and just over 60% of that of Black American children, yet the incidence of T-ALL was not remarkably different in the four groups of children, ranging between 0.31 for Nigerian, through 0.35 and 0.38, respectively, for UK and White US, to 0.40 for Black US children, thus, serving as an internal control and indicating that the reduced incidence of other subtypes was unlikely to be due to under diagnosis. The incidence of c-ALL in the presumed Caucasian UK and White American children, however, at 1.83 and 1.91 respectively, was at least ten fold, and that of American Black children, estimated at 0.70, almost three times higher than that of Nigerian children, which has been estimated at 0.18. The incidence of B-ALL among Nigerian children, estimated at 0.18 is three to nine times higher than that of among Caucasian American and UK children with rates of 0.06 and 0.02, respectively. Thus, the main differences in the incidence of ALL in the four populations is principally attributable to the differences in the incidence of non T-ALL.

### Time-space clustering of haematological malignancies

Thirty-five occurrences of time-space clustering of subtypes of haematological malignancies were observed. Twenty-eight (80%) of these were observed within Zone 1, 6 (17.1%) in Zone 2, and 1 (2.3%) in Zone 3. The highest frequency of clustering was observed with Burkitt’s lymphoma, among which 13 (93.9%) of 14 observations of clustering (involving 31 patients) occurred within Zone 1. There were four observations of AML/AMML clustering involving nine patients all in Zone 1, and 3 of ALL involving 8 patients, 2 in Zone 2 and 1 Zone 3 and none in Zone 1.

The incidence of Burkitt’s lymphoma at 1.81–3.62 was highest in Zone 1, compared to 0.21–0.42 in Zone 2 and 0 in Zone 3. The incidence of AML/AMML of was 0.41–0.42 in Zone 1, 0.18–0.37 in Zone 2 and Zone 3, while the incidence of ALL was with 0.75–1.51 highest in Zone 3 and lowest in Zone 1 at 0.25–0.50.

**Figure 1.**
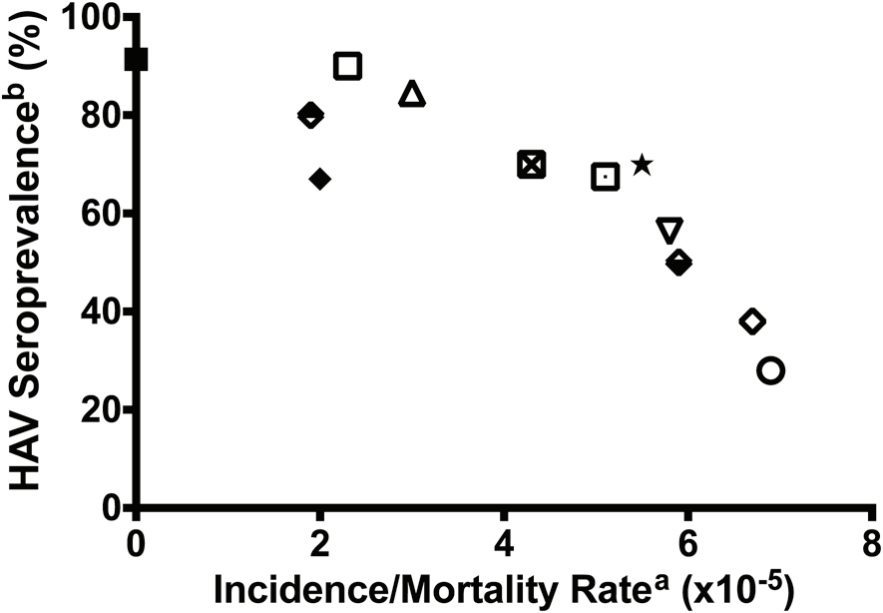
Incidence of childhood leukaemia correlated with HAV seroprevalence in the first decade of life. US Cau. = US Caucasians. Data based on Smith *et al* [40] a. Acute lymphoblastic leukaemia in 2–4 year olds. b. HAV seroprevalence rates. In developing countries of Africa, Asia, Latin America, and pre-1960 Okinawa, Japan, where age-specific variations are not observed, cited infection rates represent cross-sectional values. In the developed countries of USA and United Kingdom as well as post-1960 Okinawa, Japan, the HAV values represent infection rates in the childbearing age of 25–30 years [65].

Figure 1 illustrates the relationship between hepatitis A seroprevalence, a marker of environmental sanitation and the incidence/mortality of childhood ALL in global communities, including Ibadan, Nigeria in the mid 1980s (this study), post-World War II Okinawa (before 1960 and after 1960), the United States (Caucasians, 1976/1980), and UK (1980 and 1988/89) [40]. The apparent inverse relationship is consistent with the effect of environmental sanitation on childhood leukaemia incidence and mortality in the population.

**Key to Figure 1**

1. Solid rectangle: Leukaemia incidence in Ibadan, Nigeria as presented in this paper; HAV seroprevalence rates in Ibadan, Nigeria 1982 [66].
2. Upper closed diamond: US Caucasians 1921–1925 [65]. Infant mortality rate [67], another measure of the adequacy of public hygiene [65], has been used as a surrogate index of HAV seroprevalence rate.
3. Lower closed diamond: US Caucasians 1956–1960 [65].
4. Open diamond: US Caucasians 1976-1980 (cross-sectional HAV rate used) [65].
5. Closed diamond: US Non Caucasians 1946–1950 [65].
6. Open rectangle: Okinawa, Japan, before 1960 [65].
7. Open rectangle with a dot: Okinawa, Japan 1980–1984.
8. Standing triangle: Bangalore, India [68].
9. Inverted triangle: Philippines, Manila 1988–1992 [68].
10. X-open circle: Peru, Lima, 1990–1991 [68].
11. Black star: Columbia, Cali 1987–1991 [65, 68].
12. United Kingdom, 1988–1989 [65, 68].

## Discussion

One of the consequences of the development in recent times of monoclonal antibodies is that lymphoid neoplasias are now recognizable as heterogeneous with respect to the differentiation-associated markers present on their cells [41]. A comparison of the patterns of ALL subtypes among Nigerian children and adults as observed in this study with the observations in British, American, and Malaysian studies (Table 2) reveals a marked deficit of c-ALL in the Nigerian children. If it is correct to assume that the Malaysians and Black Americans are socio-economically intermediate between Nigerians and the Caucasian populations of the UK and United States, one could suggest an apparent correlation between the relative frequency of c-ALL in the compared populations and their socio-economic status. It also appears reasonable to suggest that the most striking differences in the epidemiology of childhood ALL in Nigeria and the socio-economically developed countries is probably due to the deficit of c-ALL and excess of B-ALL (Table 3) among Nigerians. Ramot and Magrath [42] have highlighted the possible role of economic deprivation in the aetiology of Burkitt’s lymphoma, a condition that is believed to be related to B-ALL. Low socioeconomic status also characterized the population within which BL was prevalent in Eastern Nigeria [8]. Indeed, any theory explaining the mechanism of deficit of c-ALL among African children should also be able to explain the concomitant excess occurrence of B-cell leukaemia and Burkitt’s lymphoma in the same population. Overall, the data presented in this communication are consistent with the fact that the lifestyle of socioeconomic deprivation with its hallmarks of malnutrition, sanitary deficiencies, and endemic infections, is associated with reduced incidence of c-ALL and excess of B-ALL subtypes, while the reverse is the case in high-income societies [9, 43].

In addition to the reduced incidence of c-ALL, and increased incidence of BL, the increased frequency of chloroma-associated acute myelogenous leukaemia (otherwise known as chloroleukaemia) is the another feature of childhood haematological malignancies in developing countries. With 5 of 8 cases (62.5%) of AML/AMML in under-5-year olds occurring in association with chloromas as compared to 2 of 18 (11%) in older individuals [44], chloroma-associated AML/AMML appears to be a specific disorder of childhood in Nigeria rather than a manifestation of late presentation. This form of AML/AMML presentation has been reported in various parts of the world, including Turkey [45], Egypt [46], and Central Africa [47]. In a report from South Africa, 24% of black children but no Caucasian child presented with CA-AML [48]. Chloroma-associated AML was reported in 3 of 162 cases in a United Kingdom report [35] and in 1 of 114 Caucasian children in an US report [36] thus making this form of AML presentation 28 times and 60 times more frequent in Nigerian children than in UK and in Caucasian US children, respectively [10]. This observation is most probably related to biological variations resulting from differences in contemporary lifestyles, rather than ethnicity. Thus, in Ibadan, clustering of chloroma-associated AML/AMML was observed at 2-fold increase of incidence in areas inhabited by the people of low SES lifestyle, while ALL clustered at 3-fold increased incidence in areas inhabited by medium to high SES [49], thus suggesting a role for lifestyle associated environmental influences. Such influences appear to include the effects of social deprivation, infection, and the state of hygiene [40, 42] as well as malnutrition-related thymolymphatic and acquired immunodeficiency states [50, 51]. The more recent observation from another part of Nigeria [8] on the epidemiology of BL is consistent with this observation. Lack of reporting of chloroma cases in the recent literature on paediatric malignancies from Nigeria [8, 12] would tend to suggest that the condition is either not being recognized or it may be disappearing in the area. If the latter is the case, it may mirror the course of events in Great Britain, where chloromas were believed to have been more common in the past than in more recent years [52], thus leading to the speculation that chloroma frequency will diminish in African childhood pathology with the improvement of child health in the region [53].

The epidemiological features of childhood leukaemia in the low-income parts of Africa, with the reduced incidence of ALL and the increased incidence of chloroma-associated AML, are reminiscent of the experimental observations of Ludwik Gross on the influence of environmental factors in animal leukaemogenesis. Following the underfeeding of Ak mice, Gross observed a delay in the onset of and a reduction in the rate of occurrence of virus-induced leukaemia. Thymectomy also inhibited or considerably delayed the development of lymphatic leukaemia, and frequently caused the myelogenous forms to appear later in life, often in the form of chloroleukaemia [54]. These observations have led to the hypothesis that childhood leukaemia/lymphoma epidemiology in Nigeria could be Nature’s equivalent of Ludwig Gross’s experiment, with thymo-lymphatic deficiency resulting from the combination of maternal and intra-uterine fetal malnutrition [55]. The impact of SES on early life non-neoplastic diseases has been well documented by Aizer and Currie [56].

Biological and immunological derangement also appear to be the underlying rationale for the hypothesis of ‘delayed-infection’ advanced by Greaves to explain the pathogenesis of ALL in the 2–5 year age-group in ‘modern or affluent societies’ [16], and the ‘population mixing’ hypothesis of Leo Kinle [57].

For a biological model to be plausible in explaining the epidemiologic patterns of leukaemia/lymphoma subtypes in a developing society, such as Nigeria, it should explain the three main features of childhood leukaemia and lymphoma in Nigerians, namely, reduced incidence of c-ALL subtypes, increased incidence of Burkitt’s lymphoma, and the increased incidence of chloroma-associated AML/AMML. None of the three biological models discussed earlier satisfactorily explains the epidemiological patterns of childhood leukaemia/lymphoma described in this report. The two-fold increased incidence and clustering of chloroma-associated AML/AMML in the absence of clustering of, but two-fold reduced incidence of ALL in the low SES area of Ibadan appears to be most consistent with the studies of Ludwik Gross [54], except that the model did not specify the subtypes of leukaemia/lymphoma (which had not been discovered at the time of the experiments). The hypothesis of ‘delayed infection’ [16] is consistent with the marked reduction of c-ALL subtype observed in this report, especially given the ubiquitous and prevalent relatively unhygienic status in a developing society, and hence ‘hyper-immune’ status as compared to the affluent society for which it has been construed [15]. However, this hypothesis does not explain the high incidence of B-cell lymphoma subtypes and chloroma-associated AML/AMML as described in this report. The ‘population mixing’ hypothesis of Kinle [57] is consistent with the observation of clustering of ALL in the medium and high SES areas of Ibadan, given the free intermingling in these areas of children of varied life-styles, presumably including ‘immune’ and ‘non-immune’ ones. However, this hypothesis does not explain the frequent occurrence of choroma-associated AML/AMML in Nigerian children.

The global and temporal trend in the incidence and/or mortality of ALL in various parts of the world (Figure 1) appears to correlate with the state of sanitation and hygiene within communities, thus indicating a role for sanitation-related factors globally [40]. It is unclear how or at what stage of life these environmental factors impact the human development. However, the observations correlate with observation of others as to the influence of maternal socioeconomic disadvantages and the health at birth. It is known that the fetus is particularly vulnerable to myriad health insults, some of which are preventable with appropriate health policies, including vaccination of pregnant women against influenza, supplementing food and nutrition of pregnant women through government programmes, and reduction of exposure to environmental toxins during pregnancy [56].

The observations described in this report date back a few decades. They are now being re-analysed and discussed in the light of newer development in our understanding of environmental influence on the disease processes of fetuses and children [56], including leukaemogenesis [58]. Furthermore, and in spite of a number of limiting factors, including limited reliable population figures for Ibadan, paucity of the number of cases, and the quality of the laboratory facilities and reagents available at the time of the study, it is important to observe that, in the intervening period of time, no other study from the region has addressed the issues covered in this report [8, 11, 12].

The subject of the aetiology of childhood leukaemia and lymphoma remains relevant today in all parts of the world. The lifestyles of developing societies that appear to be the underlying causes of the unique features of childhood leukaemia and lymphoma described in this report (Table 4) persist, and appear to mirror the consequences of the primordial pressures that have shaped human genetics and pathophysiology [59], apparently mediated by the macrophages, which have been described as an evolutionary ancient cell type [60]. Meanwhile, technological development, including flow cytometry for more accurate immunophenotypic cell characterization, availability of better reagents, and molecular biological methodologies for viral studies, among others, give us a unique opportunity to elucidate the mechanism of the interaction of genetic and environmental factors in childhood with the potential for new knowledge in leukaemia prevention and management.

## Conclusion

The differences in the incidence and clinical manifestations of childhood leukaemias/lymphomas between the low-income regions of Africa and the high-income regions of the world are related to the differences in lifestyles that are prevalent in these varying parts of the world. They indicate the greater role of environmental pressures on leukaemogenesis compared to that of genetic differences of ethnicity and race. It is expected that as socio-economic differences among populations even out, so also will the observed differences in the epidemiological features of childhood leukaemia/lymphoma.

## Figures and Tables

**Table 1.** Patterns of rearrangement of immunoglobin (IgH), and T-cell receptor (TCR) beta- and gamma-genes, correlated with HTLV-I Western blot serology and DNA in leukaemia and lymphoma patients.

GR Pattern										
**Serial no.**	**Diagnosis**	**Restr. Enzyme**	**GR Pattn**	**Restr. Enzyme**	**CB1**	**CB2**	**Restr. Enzyme**	**GR Pattn**	**WB**	**HTLV-I DNA**
1	ALL	Eco RI	G/G	Eco RI	DG	RG	ND	ND	ND	–
2	ALL	Eco RI	G/G	Eco RI	GG	GG	ND	ND	ND	–
		Hind III	G/G							
3	ALL	Hind III	G/G	Eco RI	GG	GG	ND	ND	ND	–
		Eco RI	G/G							
4	ALL	Hind III	G/G	Eco RI	GG	GG	ND	ND	ND	–
		Eco RI	G/G							
5	AML	Hind III	G/G	Eco RI	GG	GG	ND	ND	ND	–
		Eco RI	G/G							
6	AML	?	G/G	?	GG	GG	?	GG/GG	ND	–
7	?CLL/?ALL	Hind III	G/G	Eco RI	GG	GG	?	GG/GG	ND	–
8	B-CLL	Hind III	G/R	Eco RI	GG	GG	ND	ND	ND	–
9	B-CLL	Hind III	R1/R2	Eco RI	GG	GG	ND	ND	ND	–
		Eco RI	R1/R2							
10	B-CLL	Hind III	D/R	Eco RI	GG	GG	??	GG/GG	NEG	–
		Eco RI	D/R							
11	B-CLL	Hind III	G/G	Eco RI	GG	GG	??	GG/GG	NEG	–
12	B-CLL	Hind III Eco RI	G/G?						POS	–
		Eco RI	G/G?							
13	B-CLL	Hind III	R/G	Eco RI	GG	GG	ND	ND	POS	–
		Eco RI	R1/R2							
14	NHL-DL	Eco RI	G/G	Eco RI	GG	GG	ND	ND	ND	–
15	HD	??	GG	??	GG	GG			ND	–
16	NHL	??	G/G		GG	GG		ND	ND	–
		??	?/R		GG	GG		ND	ND	
17	BL	??	R1/R2	??	GG	GG	??	ND	ND	–
18	AML		G/G	??	GG	GG	??	ND	ND	–

Restrict.: Restriction; GR pattn: Gene rearrangement pattern; ALL: Acute lymphoblastic leukaemia; AML: Acute myelogenous leukaemia; CLL: chronic lymphocytic leukaemia; B-CLL: B-cell chronic lymphocytic leukaemia; NHL-DL: Non Hodgkin’s lymphoma diffuse large-cell type; HD: Hodgkin’s disease (lymphoma); HTLV-I: Human T-cell leukaemia/lymphoma type 1; BL: Burkitt’s lymphoma; ND: Not done; G: Germ line pattern; R: rearranged pattern; IgH: immunoglobin heavy chain.

**Table 2.** Pattern of acute lymphoblastic subtypes in Nigerians compared with those of the United States, UK, and Malaysia, including numbers of cases and frequency (%).

	Ibadan/Nigeria^*^		USA (Caucasians)^**^		United Kingdom^@^		African-Americans^***^	Malaysians^$^
Age–groups in years	<15	≥15	<15	≥15	<15	≥15	<15	<15
c-ALL	4 (22.2)	8 (38.1)	217 (74.4)	37 (47.4)	398 (73.2)	23 (54.8)	16 (55.2)	7 (50.0)
Null ALL	2 (11.1)	1 (4.8)	21 (7.2)	?	68 (12.5)	11 (26.2)	4 (13.8)	3 (21.4)
T-ALL	7 (38.9)	11 (52.4)	45 (15.5)	20 (25.6)	73 (13.5)	5 (11.9)	9 (31.0)	4 (28.6)
B-ALL	4 (22.2)	1 (4.8)	8 (2.7)	?	4 (0.7)	1 (2.4)	0 (0.0)	0 (0.0)
Unclassifiable	1 (5.6)	0 (0.0)	0 (0.0)	21 (26.9)	0 (0.0)	2 (4.8)	0 (0.0)	0 (0.0)
Total	18 (100)	21 (100)	291 (100)	78 (100)	542 (100)	42 (100)	29 (100)	14 (100)

* Ibadan, Nigeria data based on this report.

** USA (Caucasians) data based on [38]

*** African-American data based on [39]

@ United Kingdom (<15 years) data based on [37] and (≥15 years) data based on [61]

$ Malaysia data based on [62]

**Table 3.** Comparative incidence^a^ of subtypes of acute lymphoblastic leukaemia in Nigerian, United Kingdom and US American children.

Leukaemia subtypes	Nigerian	United Kingdom	USA Caucasian	African-American
ALL (all subtypes)	0.8^b^	2.61^c^	2.46^d^	1.26^d^
c-ALL	0.18^e^	1.91^f^	1.83^f^	0.70^g^
T-ALL	0.31^e^	0.35^f^	0.38^f^	0.40^g^
B-ALL	0.18^e^	0.02^f^	0.06^f^	0.0^g^

a : New cases per 100,000;

b : [14];

c : [35]; [36];

e : derived from b and data in Table 2;

f : derived from c and data in Table 2;

g : derived from d and the data in Table 2.

**Table 4.** Estimated population sizes and incidence^a^ of haemopoietic malignancies among three socio-economic groups of inhabitants of Ibadan, Nigeria (1979–1978).

		Low (SES 4 + 5)	Medium (SES 3)	High (SES 1 + 2)
% of total population^b^		75	12.5	12.5
Estimated population size^c^ ×10^3^		750–1500	125–250	125–250
Adults (53%)^d^ × 10^3^		397.5–795	66.25–132.5	66.25–132.5
Children (47%)^d^ × 10^3^		352.5–705.0	58.75–117.5	58.75–117.5
**Disease**	**Age range in years**	**[Number of cases] and incidence^a^ (×10^-5^)**		
BL	<15	[51] 1.81–3.62	[1] 0.21–0.42	[0] 0.0
ALL	<15	[8] 0.25–0.50	[3] 0.56–1.13	[4] 0.75–1.51
AML	<15	[13] 0.41–0.82	[1] 0.18–0.37	[1] 0.18–0.37
ALL	≥15	[4] 0.11–0.22	[0] 0.0	[0] 0.33–0.67
AML	≥15	[7] 0.20–0.39	[1] 0.16–0.33	[1] 0.16–0.33
CML	≥15	[11] 0.17–0.33	[2] 0.18–0.36	[2] 0.18–0.36
CLL	≥15	[14] 0.20–0.41	[1] 0.09–0.17	[1] 0.09–0.17
HD	<15	[6] 0.19–0.37	[2] 0.42–0.85	[0] 0.0
HD	≥15	[16] 0.50–1.00	[9] 1.70–3.40	[0] 0.0
NB/NHL	0–80	[25] 0.42–0.83	[3] 0.30–0.60	[2] 0.20–0.40

a Leukaemia and lymphoma incidence (cases per 100,000 per year) is based on total number of cases(in bracket) accruing over 4.5 years (July, 1978 to December 1983) for leukaemia, and over 4 years(Jan. 1979 to December 1983) for lymphoma.

b [63]

c Projected from 1963 census figures at the estimated growth rate of between 2.5% and 5.0% [64].

d World Bank [34].
